# Spatio-Temporal Variation of *Synechococcus* Assemblages at DNA and cDNA Levels in the Tropical Estuarine and Coastal Waters

**DOI:** 10.3389/fmicb.2022.837037

**Published:** 2022-03-03

**Authors:** Ting Wang, Xiaomin Xia, Jiawei Chen, Hongbin Liu, Hongmei Jing

**Affiliations:** ^1^CAS Key Laboratory for Experimental Study Under Deep-Sea Extreme Conditions, Institute of Deep-Sea Science and Engineering, Chinese Academy of Sciences, Sanya, China; ^2^Key Laboratory of Tropical Marine Bio-Resources and Ecology, South China Sea Institute of Oceanology, Chinese Academy of Sciences, Guangzhou, China; ^3^Department of Ocean Science, The Hong Kong University of Science and Technology, Kowloon, Hong Kong SAR, China; ^4^HKUST-CAS Sanya Joint Laboratory of Marine Science Research, Chinese Academy of Sciences, Sanya, China; ^5^Southern Marine Science and Engineering Guangdong Laboratory, Zhuhai, China

**Keywords:** *Synechococcus*, spatio-temporal variation, genetic diversity, gene and gene transcript, tropical marine waters

## Abstract

*Synechococcus* is a major contributor to global marine primary production. Here, its spatio-temporal variations in abundance and phylogenetic structure were studied at three stations of the South China Sea at both DNA and cDNA levels. *Synechococcus* cell abundance was lowest in March, but highest in October at two coastal stations. Its abundance was higher at the estuarine station, which reached a peak value of 1.36 × 10^5^ cells/ml in April, owing to the nitrogen nutrients discharged from the Sanya River. Gene and gene transcript abundances of four *Synechococcus* lineages, clades II, III, VIII, and S5.3, were studied by quantitative PCR, which showed that clade II was the most abundant lineage at both DNA and cDNA levels. High-throughput sequencing revealed that, at the DNA level, *Synechococcus* assemblage was dominated by clade SY4 (a novel clade defined in this study), S5.2, and clade II in the coastal waters and was dominated by freshwater/S5.2 *Synechococcus*, reaching a value up to 88.61% in June, in estuarine waters. Changes in salinity and nutrient concentration caused by seasonal monsoonal forcing and river discharge were the key determinants of the spatio-temporal variation in *Synechococcus* assemblages at the DNA level. In comparison, high dissimilation among samples at the same stations and in the same seasons leads to the imperceptible spatio-temporal variation pattern of *Synechococcus* assemblages at the cDNA level. Furthermore, co-occurrence networks disclosed that *Synechococcus* community had closer and more complex internal interactions at the cDNA level. These discrepancies highlighted the necessity to study *Synechococcus* assemblages at both DNA and cDNA levels.

## Introduction

Marine *Synechococcus* represents one of the most abundant phytoplankton in the global ocean (Flombaum et al., 2013). It is widely distributed in marine ecosystems from equatorial to polar sea waters without a latitude limit (Farrant et al., 2016). Niche models even project an increase in global cell numbers of *Synechococcus* in the future (Flombaum et al., 2013; Schmidt et al., 2020), which may attribute to its specific accommodative strategies, such as efficient harvesting of both light and iron to sustain photosynthesis (Palenik et al., 2006; Schmidt et al., 2020). On the other hand, *Synechococcus* is an important component of the marine microbial food loop and contributes significantly to the primary productivity of the world’s oceans, creating 16.7% of ocean net primary production (Iturriaga and Mitchell, 1986; Flombaum et al., 2013). As a result, *Synechococcus* attracts increasing research attention (Palenik et al., 2006; Buitenhuis et al., 2012; Flombaum et al., 2013; Schmidt et al., 2020).

Marine *Synechococcus* exhibits high genetic diversity (Farrant et al., 2016; Xia et al., 2019). Gene markers, such as the 16S rRNA gene (Mackey et al., 2017), the 16S–23S internally transcribed spacer (ITS) (Li et al., 2021; Nagarkar et al., 2021), the RNA polymerase gene (*rpo*C1) (Kent et al., 2019; Wang et al., 2021a), the nitrate reductase gene (*nar*B) (Robidart et al., 2012), the cytochrome *b*6 gene (*pet*B) (Mazard et al., 2012), and the ribulose–1,5–bisphosphate carboxylase oxygenase gene (*rbc*L) (Paerl et al., 2012), have been commonly applied to study the genetic diversity of *Synechococcus*. Among these gene markers, ITS is the locus with the most sequences available for primer design (Ahlgren and Rocap, 2012). Meanwhile, although ITS is divergent, it contains conserved regions for the design of non-degenerate primers (Ahlgren and Rocap, 2012). The *rpo*C1 gene is single copy in all known genomes of *Synechococcus* strains and displays higher genetic resolution than the 16S rRNA gene (Mühling et al., 2006). On the basis of gene markers, like the 16S rRNA gene, marine *Synechococcus* strains can be classified into three major subclusters, i.e., S5.1, S5.2, and S5.3 (Dufresne et al., 2008). S5.1 as the most diversified group contains at least 20 known lineages including clades I to XVI, CRD1/CRD2, and WPC1/WPC2. (Mühling et al., 2006; Ahlgren and Rocap, 2012; Choi et al., 2013b; Ahlgren et al., 2014). In contrast, S5.2 and S5.3 contain less recognizable lineages (Choi et al., 2013a). The distribution of different *Synechococcus* lineages varies geographically in the global ocean (Farrant et al., 2016; Sohm et al., 2016; Xia et al., 2019). Clade II is dominant in warm subtropical and tropical open ocean. In contrast, clades I and IV are largely confined in cold waters (Zwirglmaier et al., 2007, 2008). Clade III prevails in the oligotrophic, warm open ocean, whereas clades CRD1 and CRD2 are confined in sites with limited iron, high nutrient, and low chlorophyll including equatorial upwelling regions and North Pacific sites (Ahlgren et al., 2020). In addition, temporal variation in the distribution of *Synechococcus* lineages has also been observed (Jing et al., 2009; Tai and Palenik, 2009; Xia et al., 2015). For example, clade I is more abundant prior to spring, whereas clades II and III only appear in late summer and winter in the California Current (Tai and Palenik, 2009); clades II and VI are the major lineages when the summer monsoon prevailed, but clades II, IX, and miyav are the dominant clades during winter in subtropical coastal waters of Hong Kong (Xia et al., 2015).

Luhuitou Peninsula (∼109.47–109.52°E, ∼18.18–18.23°N) is located in the southernmost of Sanya city, Hainan Island in South China. It has a typical tropical monsoon climate, wet from May to October but dry from November to April. Typhoons in the wet season usually bring about 90% of the annual rainfall (Zhang C. et al., 2013). Luhuitou fringing reef (109.47°E, 18.22°N, ∼3 km long and ∼0.25–0.5 km wide) is situated in the west of the Peninsula with less disturbance from human activities since the establishment of Sanya National Coral Reefs Nature Reserve in 1990 (Meixia et al., 2008; Qiu, 2013). Xiaodong Hai (∼109.50–109.51°E, ∼18.19–18.21°N) in the east of the Peninsula also has coral reefs, but its water quality is poor in comparison with Luhuitou due to the increasing number of tourists and hotel buildings along the coast (Titlyanov et al., 2019). Sanya River located in the northern part of the Peninsula suffers from serious eutrophication and pollution in recent years and is affected by the inflow of freshwater and sanitary sewage as well (Dong et al., 2010). *Synechococcus* has been reported as an important primary producer in the surrounding waters of the Luhuitou Peninsula; however, its community composition and distribution in these different water bodies have not been well investigated yet (Ling et al., 2013).

Previous studies have mostly investigated the community composition of *Synechococcus* assemblage at the DNA level, whereas only a few studies focused on it at the cDNA level (e.g., Chung et al., 2011). Considering the fact that only metabolic active cells could be identified at the cDNA level, there must be discrepancies between these two levels. To achieve a comprehensive picture of the tempo-spatial distribution of *Synechococcus* in tropical estuarine and coastal waters, monthly samples collected from three stations with different hydrographic conditions in the tropical Sanya waters were investigated using high-throughput sequencing and real-time quantitative polymerase chain reaction (qPCR) at both DNA and cDNA levels.

## Materials and Methods

### Sampling

Monthly water samples were collected from the west side of the Luhuitou Peninsula (Stn. SL1, 109.47°E, 18.21°N) together with Xiaodong Hai (Stn. SL2, 109.50°E, 18.21°N) and Sanya River estuary (Stn. SL3, 109.50°E, 18.23°N) from June 2014 to May 2015 (Supplementary Figure 1). Stns. SL1 and SL2 are at the edge of coral reefs, representing ocean-influenced coastal stations. Stn. SL3 represents the estuarine station in the Sanya River.

In each sampling, about 5 L of seawater was sequentially filtered through 3- and 0.22-μm polycarbonate filters (47 mm, EMD Millipore, Billerica, MA, United States) for DNA/RNA extraction. Filters were put in RNAlater™ Stabilization Solution (Thermo Scientific, Wilmington, DE, United States) and stored at −80°C until further analysis. To determine the cell abundance of *Synechococcus*, 1.8 ml of seawater was fixed with 0.5%∼1% paraformaldehyde and stored at −80°C. For nutrient measurement, water samples were filtered with 0.22-μm polycarbonate filters (47 mm, EMD Millipore, Billerica, MA, United States), and the filtered liquids were stored at −20°C until analysis. For chlorophyll *a* (Chl *a*) measurement, water samples were sequentially filtered through 20-μm, 2-μm, and GF/F glass-fiber filters under low vacuum. Filters were wrapped in aluminum foil and kept frozen at −80°C.

### Measurement of Environmental Variables

Hydrographical parameters, temperature and salinity, were recorded *in situ* using an MC601 thermometer (Hangxin Technology Co., Ltd., China) and MASTER-S/MillM refractometer (ATAGO Co., Ltd., Japan), respectively. pH was determined using an ST300 portable pH meter (Ohaus Instruments Co., Ltd, China) in the laboratory. The concentration of nutrients, including nitrite (NO_2_^–^), ammonium (NH_4_^+^), total nitrogen (TN), silicate (SiO_3_^2–^), and phosphate (PO_4_^3–^), were measured with an auto-analyzer (QuAAtro, Blue Tech Co., Ltd., Tokyo, Japan). Chl *a* concentration was determined from the GF/F glass-fiber filter because its size is closer to *Synechococcus*. After extraction in 90% acetone at 4°C in the dark for 20 h, chl *a* concentration was determined using a Turner Designs fluorometer (model Trilogy 040) (Chen et al., 2009). *Synechococcus* cell abundance was counted using a Becton-Dickinson FACSCalibur flow cytometer (FCM) equipped with dual lasers of 488 and 635 nm. Forward and right-angle light scattering and four fluorescence signals were collected, saved, and analyzed using WinMDI 2.9 (Liu et al., 2014).

### DNA and RNA Extraction and cDNA Synthesis

Total DNA and RNA were extracted from the 0.22-μm filters with the PureLink Genomic DNA kit (Invitrogen, Carlsbad, CA, United States) and RNA purification kit (Invitrogen) with TRIzol^®^ Reagent, respectively. Concentrations of DNA and RNA were determined with a NanoDrop 2000C spectrophotometer (Thermo Scientific, Wilmington, DE, United States). RNA was purified with DNase I (Ambion, Life Technologies, Austin, TX, United States) and then reverse-transcribed with a SuperScript III First-strand Synthesis kit (Invitrogen). A parallel reaction without SuperScript III reverse transcription (RT) was used as a negative control (non-RT control) for the RT-PCR conducted for each sample. Residual RNA was removed by treatment with 2 U of RNase H at 37°C for 20 min. DNA and cDNA were stored at −20°C before further analysis.

### Sequencing and Real-Time Quantitative Polymerase Chain Reaction

The *rpo*C1 gene and gene transcripts were amplified using nested PCR protocols as described previously (Mühling et al., 2006). Purified amplicons were sequenced with an Illumina HiSeq PE250 sequencer (Novogene Technology Co., Ltd, China).

The ITS gene and gene transcripts of *Synechococcus* clades II, III, VIII, and S5.3 were quantified *via* a StepOnePlus Real-Time PCR System (Applied Biosystems Inc., Carlsbad, CA, United States). Each qPCR reaction comprised 7.5 μl of 2 × SYBR^®^ Premix Ex™ Taq II (TaKaRa Bio Inc., Shiga, Japan), 0.5 μM primer (Ahlgren and Rocap, 2012), 1 μl of DNA/cDNA as the template, 0.4 μl of ROX reference dye, and water to a total volume of 15 μl. The qPCR reactions and calibrations were performed following a protocol described previously (Ahlgren and Rocap, 2012). Triplicate qPCR reactions were performed for each sample with an efficiency range of ∼90%–105%, and the gene copy number was normalized to the quantity of the gene and gene transcripts. As a positive control, a linear plasmid was used, which was constructed using the amplified PCR products and a TOPO-TA vector cloning kit (Invitrogen). Both non-RT control and non-template control were always used as a negative control.

### Bioinformatics Analysis

Raw sequencing data were processed with QIIME 2 (v.2020.8.0) (Bolyen et al., 2019). The sequences were quality-controlled and then used to create amplicon sequence variants (ASVs) using DADA2 version 1.6.0 (Callahan et al., 2016) (with the q2-dada2 plugin). Diversity indices (Shannon) were calculated on the basis of the ASV data. Then, taxonomy was assigned to ASVs using the q2-feature-classifier (Bokulich et al., 2018) on the basis of a local database (Xia et al., 2017a). There were some ASVs taxonomically close to both freshwater *Synechococcus* and S5.2 (*Synechococcus* sp. CB0101) sequences, which were defined as FS/S5.2. Representative sequences with similarity to reference sequences less than 90% were defined as unclassified. Representative sequences of unclassified ASVs in top 700 ASVs were selected to construct the maximum likelihood (ML) tree with reference sequences using IQ-TREE version 1.6.12 (Nguyen et al., 2015). The best-fit model SYM + I + G4 according to the Bayesian Information Criterion with 5,000 bootstraps was set to construct the tree.

### Statistical Analyses

One-way analysis of variance with *post hoc* test least significant difference was performed to test the significance of differences in cell or gene abundances among samples at different stations in different seasons. Linear discriminant analysis effect size (LEfSe) analysis was performed to identify *Synechococcus* lineages with significant differences in relative abundance among samples at different stations in different seasons (Segata et al., 2011). Redundancy analysis (RDA) was performed with package vegan in R Language (version 3.4.2), to estimate correlations between the distributions of *Synechococcus* lineages and environmental variables. The proportion of different phylogenetic groups was Hellinger transformed, environmental variables were logarithm transformed, and the effects of collinearity (variance inflation factor > 10) were removed. The statistical significance of an explanatory variable added in the course of forwarding selection was tested with the Monte Carlo permutation test (9,999 permutations, *p* < 0.05). Box plots were drawn using Origin version 9.65. Co-occurrence networks were constructed on the basis of the Spearman’s correlation matrixes (*p* < 0.05) of ASVs at DNA and cDNA levels, respectively. The Fruchterman–Reingold algorithm in Gephi was used to lay out the networks (Bastian et al., 2009).

### Data Available

All the *rpo*C1 gene sequences obtained from this study have been deposited in the National Center for Biotechnology Information Sequence Read Archive under the accession number PRJNA753587.

## Results

### Hydrographic Conditions

**FIGURE 1:**
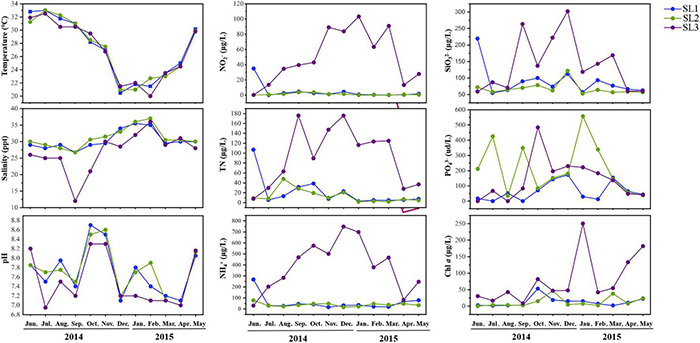
Environmental parameters of the three stations during the studied period.

Coastal Stns. SL1 and SL2 without direct river input exhibited similar hydrographic and trophic conditions (Figure 1). Salinity was high but Chl *a* and nutrient concentrations (i.e., of TN, NH_4_^+^, NO_2_^–^, and SiO_3_^2–^) were low at these two stations throughout the studied period. PO_4_^3–^ concentration was seasonally fluctuant and was higher at Stn. SL2 with stronger human activities effect. On the contrary, estuarine Stn. SL3 was strongly influenced by freshwater discharge from the Sanya River, with low salinity and Chl *a* concentrations. Seasonally, temperature was significantly higher in summer (June to August) (*p* < 0.01), whereas salinity was significantly higher in winter (December to February) at all three stations (*p* < 0.05). Besides, higher nutrient concentrations including TN, NH_4_^+^, NO_2_^–^, PO_4_^3–^, and SiO_3_^2–^ were usually detected in winter.

### Cell and Gene Abundance

**FIGURE 2:**
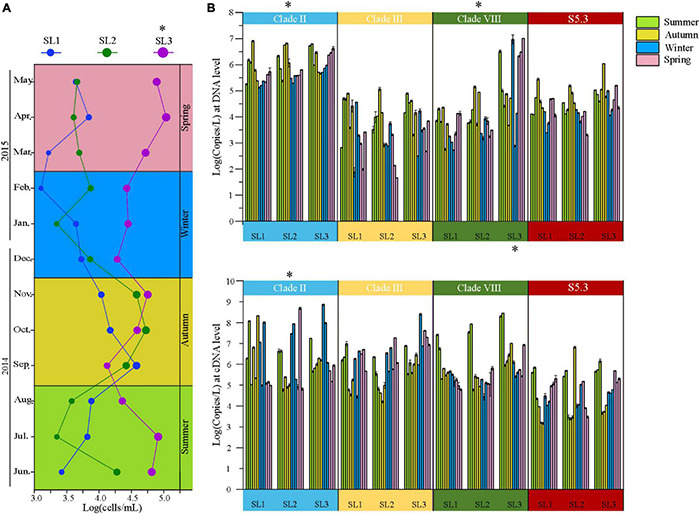
**(A)** Temporal variation of *Synechococcus* cell abundance at three stations. **(B)** Gene (top panel) and gene transcript (bottom panel) abundance of *Synechococcus* lineages at three stations. * – *p* < 0.05; Summer, June to August; Autumn, September to November; Spring, December to February; Winter, March to May.

The cell abundance of *Synechococcus* measured by FCM ranged from 1.59 × 10^3^ to 1.36 × 10^5^ cells/ml at the three sampling stations. The average cell abundance was 6.16 × 10^4^ cells/ml at estuarine Stn. SL3, which was significantly higher than that at the coastal Stns. SL1 (1.09 × 10^4^ cells/ml) and SL2 (1.80 × 10^4^ cells/ml) (*p* < 0.01) (Figure 2A). Coastal stations had higher *Synechococcus* abundance in Autumn (September to November), reaching peak values of 4.73 × 10^4^ cells/ml in September at Stn. SL1 and 6.62 × 10^4^ cells/ml in October at Stn. SL2, respectively. In comparison, estuarine Stn. SL3 had higher *Synechococcus* abundance in Spring (March to May) and Summer, reaching a value up to 1.36 × 10^5^ cells/ml in April.

The gene abundance of *Synechococcus* S5.1 clades II, III, and VIII as well as S5.3 ranged from 4.79 × 10^1^ to 1.06 × 10^7^ copies/L (Figure 2B). Generally, *Synechococcus* clade II had the highest gene abundance, reaching 1.73 × 10^6^ copies/L on average at three stations. The gene abundance of clade VIII was also high, with an average of 8.25 × 10^5^ copies/L at three stations. Comparatively, that of clade III and S5.3 was only 1.81 and 8.07 × 10^5^ copies/L, respectively. Spatially, we found significantly higher gene abundances of clade VIII at Stn. SL3 than that at the two coastal stations. Seasonally, clades II and S5.3 had higher gene abundance from September to November, whereas clade III was more abundant from June to August than that in other months (Supplementary Figure 2).

As for the gene transcript, the abundance of four detected lineages was from 1.50 × 10^3^ to 7.27 × 10^8^ copies/L (Figure 2B). The gene transcript abundance of clade II was also significantly higher than other three lineages at three stations (*p* < 0.05). Higher gene transcript abundance of clade III was found from March to May, whereas clade VIII from June to August. Generally, the metabolic activity of clade III was the highest, especially from March to May (*p* < 0.05), as indicated by the cDNA/DNA ratio (Supplementary Figure 2). Significantly higher metabolic activity of the clade VIII was also detected from June to August (*p* < 0.05).

### Community Diversity and Phylogeny

High-throughput sequencing generated ∼1.6 million high-quality reads from all 36 samples (Table 1). These reads were further assigned to 8,228 ASVs. Samples at the DNA level had much higher ASV number and Shannon index than those at the cDNA level. At the DNA level, ASV number ranged from 145 (SL3, June) to 490 (SL1, August) and Shannon index ranged from 4 (SL1, January) to 5.23 (SL1, August). Higher ASV number and Shannon index usually appeared at coastal Stns. SL1 and SL2. Seasonally, ASV number and Shannon index were higher in summer at coastal stations, whereas in winter at the estuarine station. On the other hand, at the cDNA level, ASV number ranged from 59 (SL1, December) to 523 (SL2, February) and Shannon index ranged from 0.83 (SL2, January) to 5.29 (SL3, January). Higher ASV number was found at Stn. SL2 (205 on average), whereas higher Shannon index was shown at Stn. SL3 (3.06 on average). Seasonally, both higher ASV number and Shannon index were found in winter at three stations.

**TABLE 1:** Results of *rpo*C1 amplicon sequencing at DNA and cDNA levels.

Station	Month	DNA level				cDNA level			
		High-quality sequence	ASVs (100%)	Shannon	Goods coverage	High-quality sequence	ASVs (100%)	Shannon	Goods coverage
SL1	June	29,164	345	4.96	0.99	67,292	86	2.09	0.99
	July	20,252	270	4.86	0.99	63,927	96	2.20	0.99
	August	40,891	490	5.23	0.99	66,484	211	1.69	0.99
	December	29,239	231	4.53	0.99	59,494	59	1.03	0.99
	January	29,516	149	4.00	0.99	59,026	165	4.28	0.99
	February	55,144	350	4.73	0.99	60,380	333	3.41	0.99
SL2	June	19,753	264	4.97	0.99	49,953	90	2.84	0.99
	July	28,693	284	4.78	0.99	66,196	78	1.97	0.99
	August	46,233	389	5.07	0.99	59,334	193	3.24	0.99
	December	28,787	198	4.40	0.99	63,018	256	4.78	0.99
	January	34,213	202	4.12	0.99	62,735	90	0.83	0.99
	February	47,472	480	5.20	0.99	62,681	523	4.13	0.99
SL3	June	17,465	145	4.43	0.99	60,400	65	1.95	0.99
	July	17,777	195	4.58	0.99	53,038	81	2.57	0.99
	August	43,030	299	4.32	0.99	56,069	141	2.96	0.99
	December	17,452	164	4.38	0.99	61,706	101	2.98	0.99
	January	23,054	146	4.26	0.99	47,148	495	5.29	0.99
	February	39,825	379	5.15	0.99	60,772	161	2.59	0.99

Four novel clades, SY1 to SY4, belonging to *Synechococcus* S5.1 were identified from the ML phylogenetic tree (Supplementary Figure 3). Both clades SY1 and SY2 contained 15 ASVs, which were phylogenetically closed to clades CRD2 (average phylogenetical distances were 13.4% and 12.4%, respectively). Clade SY3, which included only one ASV, was phylogenetically closed to some rare clades, such as WPC1 (average phylogenetical distance 10.0%). Finally, 24 ASVs fell into the last novel clade, SY4, which had a close phylogenetic distance to clade XVI (14.1%).

### Spatio-Temporal Variations of *Synechococcus* Community Composition

**FIGURE 3:**
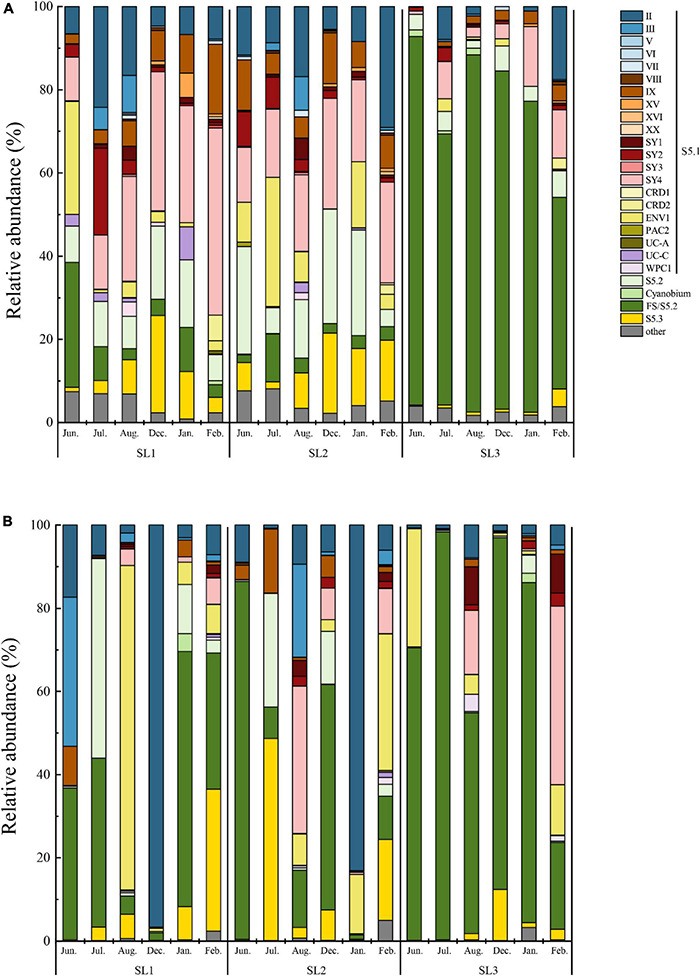
Community structure of *Synechococcus* assemblages at DNA **(A)** and cDNA **(B)** levels.

At the DNA level, a total of 25 *Synechococcus* lineages were detected at three stations, including the four novel clades (Figure 3A). The community structure showed a similar pattern at coastal Stns. SL1 and SL2. The most dominant lineage was clade SY4, followed by S5.2 and clade II at the coastal waters. Among them, clade SY4 was more abundant in winter at both two stations. Especially at Stn. SL1, clade SY4 occupied 35.49% of the total *Synechococcus* community in winter. S5.2 reached a peak of 16.26% in January at Stn. SL1, and 27.54% in December at Stn. SL2. Clade II had a higher proportion in July (24.18%) at Stn. SL1 and in February (29.08%) at Stn. SL2. On the contrary, the community structure at estuarine Stn. SL1 was different to that at coastal stations at the DNA level. FS/S5.2 was the absolute predominant lineage, reaching 73.61% on average in all estuarine samples. Besides, six clades, including clades UC-A, PAC2, CRD2, SY3, XX, and XVI, were absent in the estuarine station at the DNA level.

At the cDNA level, only a total of 22 *Synechococcus* lineage were detected at three stations, in which clades PAC2, CRD2, and XX were absent (Figure 3B). Furthermore, clade XVI was not detected at coastal Stns. SL1 and SL2, whereas clades V and VII were not detected at estuarine Stn. SL3. The dominant lineage changed to FS/S5.2, followed by clades II and ENV1 at the coastal waters at the cDNA level. The average percentage of FS/S5.2 was only 29.20% in all coastal samples, whereas, it was increased in some months, such as in June at Stn. SL2 (86.13%). Similarly, clade II had high proportions in December at Stn. SL1 (96.67%) and in January at Stn. SL2 (83.10%), but kept low proportions (commonly less than 10%) in other samples. Clade ENV1 had high relative abundance in August at Stn. SL1 (77.92%). Although clade SY4 dominated *Synechococcus* communities in the coastal stations at the DNA level, it had a low relative abundance at the cDNA level, except in August at SL2. Compared to the wide distribution of S5.2 at the DNA level, S5.2 was only found with high proportions in a few samples of coastal stations at the cDNA level. On the other hand, FS/S5.2 was also the absolutely predominant lineage, reaching 68.10% on average in all estuarine samples at the cDNA level, which was lower than that at the DNA level. Furthermore, compared to that at the DNA level, a higher abundance of clade SY4 in February and clade ENV1 in June was found at Stn. SL3 at the cDNA level.

**FIGURE 4:**
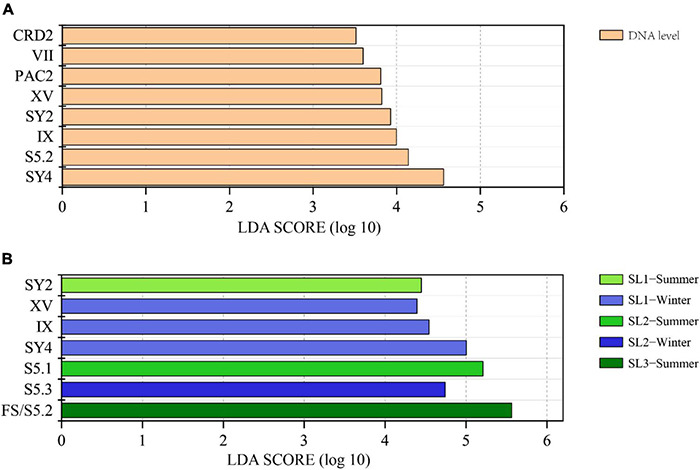
**(A)** LEfSe analysis showing the Linear Discriminant Analysis (LDA) score between DNA and cDNA levels. **(B)** LEfSe analysis showing the LDA score among stations and between seasons at the DNA level.

LEfSe analysis demonstrated that there were eight differential lineages between DNA and its corresponding cDNA, and all of them were with higher proportions at the DNA level (Figure 4A). Significantly differential lineages among stations or seasons were detected only at the DNA level (*p* < 0.05) (Figure 4B). At the DNA level, clade SY2 was the differential lineage that had significantly higher relative abundance in summer at Stn. SL1; Clades XV, IX, and SY4 had significantly higher relative abundance in winter at Stn. SL1; the total percentage of each clade of S5.1 was significantly higher in summer at Stn. SL2; S5.3 had significantly higher relative abundance in winter at Stn. SL2; and FS/S5.2 had significantly higher relative abundance in summer at estuarine Stn. SL3.

### Environmental Effects

**FIGURE 5:**
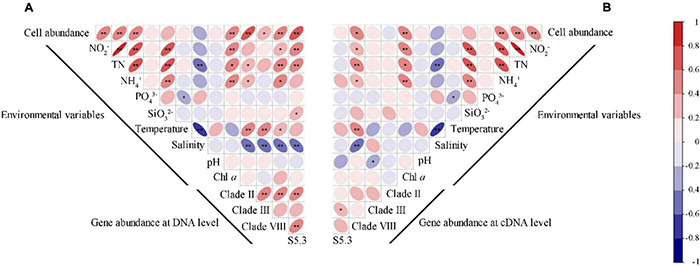
Spearman correlations between the gene **(A)** and gene transcript **(B)** abundance of *Synechococcus* lineages with *Synechococcus* cell abundance and environmental variables. * – *p* < 0.05; ** – *p* < 0.01.

Spearman’s correlation analysis revealed that *Synechococcus* cell abundance was positively correlated with the concentrations of NO_2_^–^, TN, NH_4_^+^, and Chl *a* (*p* < 0.01) (Figure 5A). Gene abundances of the four *Synechococcus* lineages significantly correlated with most environmental variables (*p* < 0.05), including NO_2_^–^, TN, NH_4_^+^, temperature, and salinity. The correlations among gene abundances and salinity were always negative, but correlations among gene abundances and other environmental variables were usually positive. Only gene abundance of S5.3 was significantly correlated with SiO_3_^2–^, and no gene abundance was significantly correlated with pH and Chl *a*. In comparison, there were less significant correlations between gene transcription abundances and environmental variables (Figure 5B). It was only found that gene transcript abundance of clade VIII was correlated with the concentrations of NO_2_^–^, TN, NH_4_^+^, temperature, and salinity, and that of clade III was correlated with pH.

**FIGURE 6:**
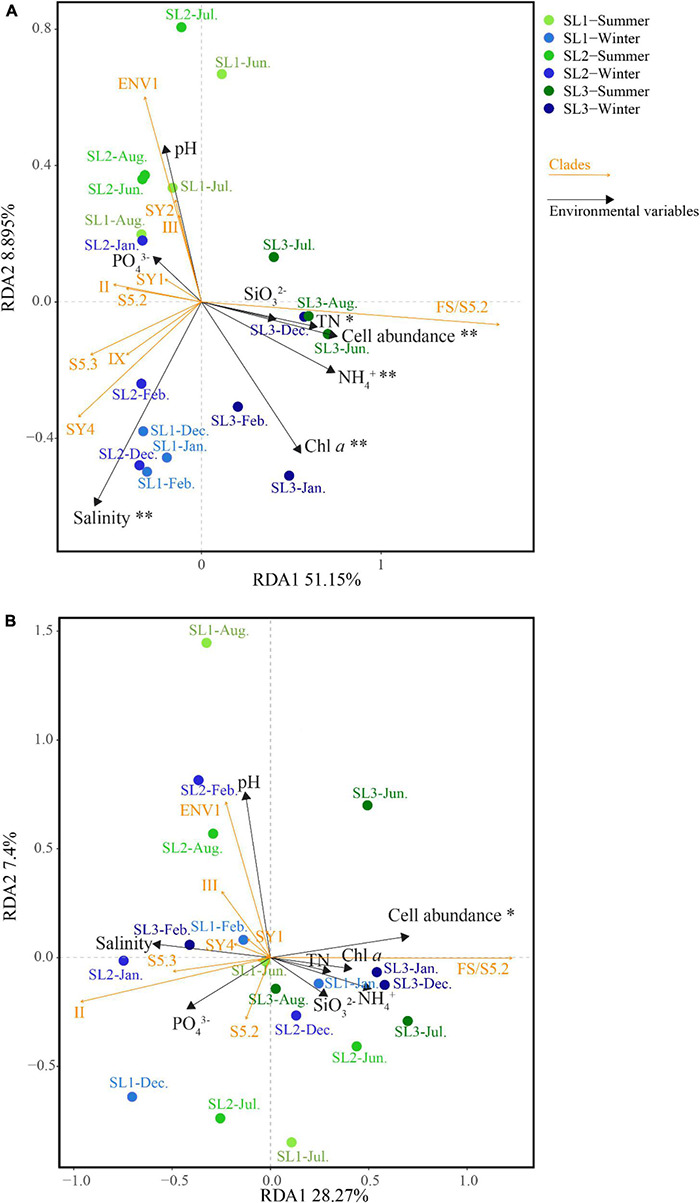
Correlation plots based on the RDA revealing the relationship among stations, environmental variables, and *Synechococcus* lineages at DNA **(A)** and cDNA **(B)** levels. * – *p* < 0.05; ** – *p* < 0.01.

At the DNA level, the first two axes of RDA on the basis of relative abundances of *Synechococcus* lineages with associated environmental parameters together explained 60.05% of the total variance (Figure 6A). It demonstrated that salinity (*p* < 0.01), cell abundance (*p* < 0.01), Chl *a* (*p* < 0.01), as well as concentrations of TN (*p* < 0.05) and NH_4_^+^ (*p* < 0.01) were the key environmental parameters that significantly influence the community structure of *Synechococcus* assemblage at the DNA level. Samples at coastal Stns. SL1 and SL2 were clustered on the left side, separated from those at estuarine Stn. SL3. Seasonally, the samples in summer were separated from those in winter, no matter at estuarine or coastal stations. Comparatively, the first two axes of RDA together only explained 35.67% of the total variance at the cDNA level (Figure 6B). It demonstrated that cell abundance was the only key environmental parameter that significantly influences the community structure at the cDNA level (*p* < 0.05). No obvious clustering was observed at the cDNA level.

### Network Analyses

**FIGURE 7:**
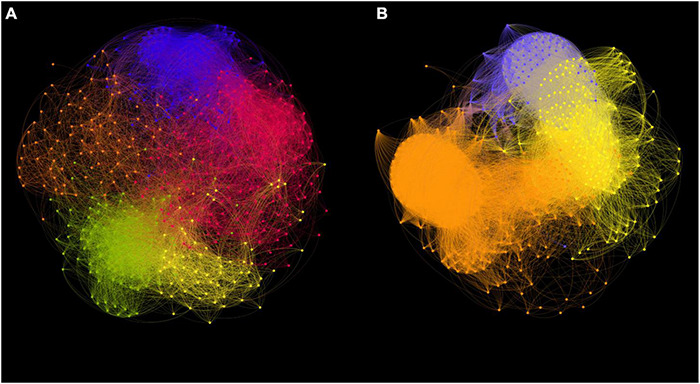
Co-occurrence network analysis of *Synechococcus* communities based on ASVs at DNA **(A)** and cDNA **(B)** levels. Nodes in networks represent ASVs. The modules are represented by different colors. The connections represent Spearman’s significant (*p* < 0.05) correlations.

Co-occurrence networks were constructed to reveal the ecological interactions among ASVs in *Synechococcus* communities at both DNA (Figure 7A) and cDNA (Figure 7B) levels. There were 523 nodes and 14,995 edges (average degree of 59.34) and 440 nodes and 26,555 edges (average degree of 122.71) in networks of S*ynechococcus* communities at DNA and cDNA levels, respectively (Supplementary Table 1). Positive correlations occupied 89.28% and 86.91% of total correlations in S*ynechococcus* communities at DNA and cDNA levels, respectively. Topological indices, including average path length, modularity, and the number of sub-communities, were higher in the network at the DNA level than those at the cDNA level. However, S*ynechococcus* community at the cDNA level has a higher clustering coefficient than that at the DNA level. Depending on closeness centrality scores, keystone ASVs in networks were identified. The top three keystone ASVs were all affiliated in S*ynechococcus* FS/S5.2 (values ranged from 0.53 to 0.54) at the DNA level, whereas those were affiliated in *Synechococcus* lineages of S5.3, clade SY3, and WPC1, at the cDNA level (values were all 0.65).

## Discussion

### Variation of *Synechococcus* Cell Abundance Between Coastal and Estuarine Stations and Controlling Factors

*Synechococcus* cell abundance ranged from 10^3^ to 10^4^ cells/ml at the coastal stations (Figure 2A). It is lower than those in temperate or subtropic coastal waters, such as Bohai coastal marine ranch (Li et al., 2021), Hong Kong coastal waters (Liu et al., 2014), coastal western subtropical Pacific (Tsai et al., 2006), and Southern California coast (Nagarkar et al., 2021). However, it is consistent with those in other tropical coastal waters, such as the equatorial eastern Indian Ocean (Wei et al., 2019) and coastal waters of the Philippines (Southeast China Sea) (Agawin et al., 2003). This phenomenon may be attributed to changes in latitude. Previous studies also unveiled strong variations of *Synechococcus* abundance with latitude, which found the peak abundance located around 45°N in the world ocean (Flombaum et al., 2013) and around 33.5°N in the marginal seas of northwestern Pacific Ocean (Xia et al., 2017b). In comparison, we found highly abundant *Synechococcus* at the estuarine station, especially more than 10^5^ cells/ml in April and July, which is probably due to the eutrophic environment in estuarine waters caused by the discharge of freshwater and sanitary sewage. Many previous studies have shown that *Synechococcus* cell abundance is the highest in summer and the lowest in winter because the temperature changes the relationship between *Synechococcus* growth and its grazing mortality (Paerl et al., 2012; Chen et al., 2020; Wang et al., 2021b). However, our result showed that *Synechococcus* cell abundance reached the highest in autumn in tropical waters. The possible reason is that, in summer, the growth of *Synechococcus* was suppressed by high temperature (> 30°C) and low-nutrient concentrations, whereas, in winter, the growth was limited by low temperature.

Correlation analyses showed that *Synechococcus* abundance was positively influenced by concentrations of Chl a and nitrogen sources, including TN, NO_2_^–^, and NH_4_^+^ (Figure 5). In comparison, previous studies suggested that the cell abundance of *Synechococcus* was mainly controlled by temperature and nutrients in many sea areas (Moisan et al., 2010; Wang et al., 2011; Xia et al., 2017b). Considering the fact that the study area is geographically located in the tropics with high temperatures, the non-significant correlation between temperature and cell abundance is reasonable. A positive correlation with Chl a concentration indicated the high biomass contribution of *Synechococcus* in the tropical waters (Agawin et al., 2003). Nitrogen sources have also been considered as an important factor affecting the *Synechococcus* distribution in other subtropical (Qiu et al., 2010; Zhang X. et al., 2013) and tropical (Rajaneesh and Mitbavkar, 2013) estuarine waters. It may be attributed to the nitrogen demand of phycobilisome-based light-harvesting system and siderophore transport systems of *Synechococcus* (Agirbas et al., 2015; Schmidt et al., 2020).

### High *Synechococcus* Diversity and Novel Lineages in Tropical Waters

According to high-throughput sequencing of the *rpo*C1 gene and gene transcript, we detected 25 and 22 lineages representing *Synechococcus* S5.1, S5.2, S5.3, FS/S5.2, and *Cyanobium* at DNA and cDNA levels, respectively (Figure 3). In comparison, previous studies found about 6 to 14 lineages in most world’s sea areas, such as Sargasso Sea (Ahlgren and Rocap, 2006), Chesapeake Bay (Chen et al., 2006), the East China Sea and the East Sea (Choi et al., 2013b), Gulf of Aqaba (Post et al., 2011), and Yellow Sea (Wang et al., 2021a). More lineages were identified in subtropical estuarine waters but are still less than those found in this study. For example, a total of 17 *Synechococcus* lineages were reported in the estuarine waters of Hong Kong by sequencing the same gene (*rpo*C1) used in this study (Xia et al., 2015). The extraordinary high *Synechococcus* diversity of our results is in part from the difference in detection method but also suggests that tropical waters are one of the regions with the highest *Synechococcus* diversity in the world.

We defined four novel clades of *Synechococcus* in the studied area (Supplementary Figure 3). Lineages restricted in narrow regions are considered as an indicator of specific marine environmental conditions, so these four clades are more indicative of typical environmental features in tropical estuaries and coasts (Sohm et al., 2016). Phylogenetically, clade SY4 was close to clade XVI that occurred in ecotone sea areas with intermediate conditions (Sohm et al., 2016; Xia et al., 2019), such as East China Sea (Choi and Noh, 2009), Red Sea (Fuller et al., 2003), and Mediterranean (Mella-Flores et al., 2011) (Supplementary Figure 3). Strains from clade XVI are capable of chromatic adaptation and are not deficient in the utilization of NO_3_^–^ or NO_2_^–^ (Ahlgren and Rocap, 2006). Consistently, clade XVI was a minor group, rarely found in our samples (only occupied 0.67% on average of total samples). However, clade SY4 was one of the most abundant clades in the studied tropical waters (Figure 3). It has significantly higher proportions at Stn. SL1 in winter than other samples at the DNA level and was mostly affected by salinity (*p* < 0.05) (Figures 4, 6). The difference of relative abundance and ecophysiology between clades SY4 and XVI verified that phylogenetic proximity in *Synechococcus* lineages does not mean their similarity in geographical preference and niche adaptation (Dufresne et al., 2008).

### Variations in Community Structure of *Synechococcus* Assemblages in Tropical Waters

Spatio-temporal variation of *Synechococcus* assemblages in studied tropical waters was observed at the DNA level (Figures 3, 4). Spatially, *Synechococcus* assemblage at estuarine Stn. SL3 differed greatly from that at coastal stations (Figure 6). Affected by the discharge of eutrophic freshwater, the tropical estuary of Sanya River was predominated by FS/S5.2. Similarly, the dominance of freshwater *Synechococcus* in estuarine waters of other regions was revealed (Xia et al., 2017a). Other studies reported the prevalence of S5.2 in estuarine waters, such as Hong Kong water (Xia et al., 2015), Pearl River estuary (Xia et al., 2017a), and Baltic Sea brackish waters (Celepli et al., 2017). However, we found that euryhaline S5.2 occupied a lower proportion at Stn. SL3 (4.37% on average) than at coastal stations (14.20% on average). Considering that the method of high-throughput sequencing only provides information of relative abundance, this phenomenon may be because S5.2 was overwhelmed by the high abundance of other lineages at Stn. SL3. Besides, it may also be caused by the ambiguity of FS/S5.2. In addition, higher gene abundances of clade VIII were detected at estuarine Stn. SL3 (Figure 2B). Although some studies considered clade VIII was specifically adapted to hypersaline waters (Dufresne et al., 2008; Huang et al., 2012), our correlation analysis showed its negative relations with salinity. Other studies supported our result, which found phycocyanobilin-only clade VIII were distributed in estuarine waters with high nutrient levels, high turbidity, and low salinity (Six et al., 2007; Xia et al., 2015). Seasonal variation of community structure was mainly detected at coastal Stns. SL1 and SL2, which was greatly affected by salinity (Figure 6). This may be attributed to the appearance of low salinity in the surface layers of sea waters due to a large amount of rainfall that occurs during the summer monsoon (Dong et al., 2010). Similar seasonal variation of *Synechococcus* assemblages caused by monsoon was also observed in the subtropical coastal waters of Hong Kong (Jing et al., 2009). In comparison, high seasonal and inter-station dissimilarities at the cDNA level resulted to imperceptible spatio-temporal variation pattern. At the cDNA level, *Synechococcus* lineage FS/S5.2 was found to be dominant in some samples at the coastal stations. Especially, its relative abundance reached 86.13% in June at Stn. SL2 at the cDNA level (Figure 3B). The sampling area has a typical tropical monsoon climate, which is wet from May to October. According to the weather record, we found that strong rainfall occurred before and even on the sampling day from June to August. Consequently, besides the imprecision in separating FS/S5.2, this unusual phenomenon may be attributed to the active freshwater *Synechococcus*, which could be temporarily brought from adjacent estuarine areas due to the increase of precipitation.

### Discrepancies in *Synechococcus* Assemblages Between DNA and cDNA Levels

Disparities between DNA- and cDNA-based communities have been reported by many studies (Steven et al., 2017; Barreto et al., 2021). The reason may be that the DNA-based analysis only represents the presence of microbial organisms in the environmental samples, but it does not imply that the corresponding species are metabolically active (Gill et al., 2017). DNA can be retained in the environment after cell death, whereas cDNA is short-lived and usually related to active growth and cell activity (Ramos et al., 2000). Consequently, simultaneous study and comparison of both components can unveil abundant and inactive, as well as rare but highly active populations (Steven et al., 2017; Barreto et al., 2021). In this study, discrepancies in gene abundance, diversity, and composition of *Synechococcus* assemblages between DNA and cDNA levels were observed as well. The diversity index of *Synechococcus* community at the cDNA level was much lower than that at the DNA level (Table 1), indicating that the metabolically active *Synechococcus* lineages constitute only a fraction of the total assemblages. This phenomenon is also found in microbial communities from different environments, reflecting that DNA-based libraries can recover a broader range of active, dormant, and even dead populations, whereas cDNA-based libraries can only detect active cells (Salgar-Chaparro and Machuca, 2019; Barreto et al., 2021). Meanwhile, there were at least three lineages that only appeared in samples at the DNA level (Figure 3A) and five lineages that had higher relative abundance in samples at the DNA level (Figure 4A). This decoupling could be explained by a response to changes in environmental conditions. *Synechococcus* have the ability to enter the dormant stage in adverse environments (Fucich and Chen, 2020). In addition, highly abundant but inactive *Synechococcus* could be brought by runoff or ocean currents from other areas or upwelled from deeper waters and might have low ecological relevance (Paerl et al., 2011; van den Engh et al., 2017).

The co-occurrence network analysis was selected to determine the discrepancies in interactions within *Synechococcus* communities at DNA and cDNA levels (Figure 7). *Synechococcus* does not exist in isolation in various ecological environments but forms complex ecological interactions with each other or other taxa (Christie-Oleza et al., 2015). The high modularity values of the two networks suggested that a modular structure existed in *Synechococcus* communities at both DNA and cDNA levels (Supplementary Table 1) (Newman, 2006). The network at the cDNA level had more edges but fewer nodes than that at the DNA level, and the average degree of the network at the cDNA level was more than twice as high as that of the network at the DNA level, indicating that there were more intricate connections among the ASVs in *Synechococcus* community at the cDNA level. Furthermore, the network at the cDNA level had a higher clustering coefficient but lower average path length than that at the DNA level, consequently exhibiting more “small-world” properties (more intense information dissemination and exchange ability) (Watts and Strogatz, 1998). The highly connected nodes in co-occurrence networks are considered as “keystone nodes” (Steele et al., 2011). Although FS/S5.2 had high relative abundance in some samples at the DNA level, they were not identified as keystone lineages in our network analysis. This is because the networks were constructed on the basis of all collected samples and FS/S5.2 was rare in some coastal samples. Besides, at the cDNA level, minor S*ynechococcus* lineages, S5.3, clade SY3, and WPC1, played a critical role in maintaining the structure steady of a *Synechococcus* community and has a greater impact on its community than that expected regardless of its relative abundance or total biomass at the cDNA level (Cottee-Jones and Whittaker, 2012).

This is the first study to compare the *Synechococcus* composition between cDNA and DNA levels. The results illustrate differences between total (DNA) and active (cDNA) *Synechococcus* communities in the tropical sea areas. A single method cannot fully reveal the phylogenetic diversity and actively functioning *Synechococcus* communities. *Synechococcus* lineages with low abundance at DNA level but relatively high proportion at cDNA level represent those numerically less abundant but are more active in playing ecological functions, e.g., carbon fixation. It is important to reveal which *Synechococcus* lineages play a key ecological role in different ecosystems, and study at the cDNA level may better reflect their real conditions.

## Conclusion

Spatio-temporal distribution patterns of *Synechococcus* in the tropical waters were investigated using qPCR and high-throughput sequencing at both DNA and cDNA levels. We found that nitrogen nutrients discharged from the Sanya River were the main factors causing the increase of *Synechococcus* cell and gene abundance in estuarine waters relative to coastal waters. Unprecedentedly, high diversity and four novel clades of *Synechococcus* were revealed in studied tropical waters. However, it is difficult to determine their physiological characters due to the lack of representative isolates. In addition, discrepancies in the diversity, composition, and interaction of *Synechococcus* assemblages between DNA and cDNA levels were illustrated. *Synechococcus* assemblage exhibited apparent spatio-temporal composition patterns and was strongly influenced by environmental variables at the DNA level. In comparison, at the cDNA level, high dissimilation among samples in the same season was detected. Furthermore, *Synechococcus* assemblage at the cDNA level was rarely correlated with environmental variables but had closer and more complex internal interactions in the co-occurrence network, which may imply the importance of biological effects on the spatio-temporal variation of active *Synechococcus* assemblages. Our result suggested that *Synechococcus* communities at both DNA and cDNA levels should be taken into account to better understand the dynamics of community structure and ecological functions of *Synechococcus* in future studies.

## Data Availability Statement

The datasets presented in this study can be found in online repositories. The names of the repository/repositories and accession number(s) can be found below: https://www.ncbi.nlm.nih.gov/, PRJNA753587.

## Author Contributions

HL and HJ conceived and designed the experiments, contributed reagents, materials, and analysis tools. HJ and XX performed the experiments. TW, XX, and JC analyzed the data. TW wrote the manuscript. XX, JC, HL, and HJ contributed writing and analysis guidance. JC contributed database. All authors contributed to the article and approved the submitted version.

## Conflict of Interest

The authors declare that the research was conducted in the absence of any commercial or financial relationships that could be construed as a potential conflict of interest.

## Publisher’s Note

All claims expressed in this article are solely those of the authors and do not necessarily represent those of their affiliated organizations, or those of the publisher, the editors and the reviewers. Any product that may be evaluated in this article, or claim that may be made by its manufacturer, is not guaranteed or endorsed by the publisher.

## References

[B1] Agawin N. S. Duarte C. M. Agustı S. McManus L. (2003). Abundance, biomass and growth rates of *Synechococcus* sp. in a tropical coastal ecosystem (Philippines, South China Sea). *Estuar. Coast. Shelf Sci.* 56 493–502. 10.1016/s0272-7714(02)00200-7

[B2] Agirbas E. Martinez-Vicente V. Brewin R. J. Racault M. F. Airs R. L. Llewellyn C. A. (2015). Temporal changes in total and size-fractioned chlorophyll-*a* in surface waters of three provinces in the Atlantic Ocean (September to November) between 2003 and 2010. *J. Mar. Syst.* 150 56–65.

[B3] Ahlgren N. A. Rocap G. (2006). Culture isolation and culture-independent clone libraries reveal new marine *Synechococcus* ecotypes with distinctive light and N physiologies. *Appl. Environ. Microbiol.* 72 7193–7204. 10.1128/AEM.00358-06 16936060 PMC1636174

[B4] Ahlgren N. A. Rocap G. (2012). Diversity and distribution of marine *Synechococcus*: multiple gene phylogenies for consensus classification and development of qPCR assays for sensitive measurement of clades in the ocean. *Front. Microbiol.* 3:213. 10.3389/fmicb.2012.00213 22723796 PMC3377940

[B5] Ahlgren N. A. Belisle B. S. Lee M. D. (2020). Genomic mosaicism underlies the adaptation of marine *Synechococcus* ecotypes to distinct oceanic iron niches. *Environ. Microbiol.* 22 1801–1815. 10.1111/1462-2920.14893 31840403

[B6] Ahlgren N. A. Noble A. Patton A. P. Roache-Johnson K. Jackson L. Robinson D. (2014). The unique trace metal and mixed layer conditions of the Costa Rica upwelling dome support a distinct and dense community of *Synechococcus*. *Limnol. Oceanogr.* 59 2166–2184. 10.4319/lo.2014.59.6.2166

[B7] Barreto M. M. Ziegler M. Venn A. Tambutté E. Zoccola D. Tambutté S. (2021). Effects of ocean acidification on resident and active microbial communities of *Stylophora pistillata*. *Front. Microbiol.* 12:707674. 10.3389/fmicb.2021.707674 34899619 PMC8656159

[B8] Bastian M. Heymann S. Jacomy M. (2009). “Gephi: an open source software for exploring and manipulating networks,” in *Proceedings of the International AAAI Conference on weblogs and social media: San Jose, California.* Available online at: https://gephi.org/publications/gephi-bastian-feb09.pdf (December 6, 2021).

[B9] Bokulich N. A. Kaehler B. D. Rideout J. R. Dillon M. Bolyen E. Knight R. (2018). Optimizing taxonomic classification of marker-gene amplicon sequences with QIIME 2’s q2-feature-classifier plugin. *Microbiome* 6:90. 10.1186/s40168-018-0470-z 29773078 PMC5956843

[B10] Bolyen E. Rideout J. R. Dillon M. R. Bokulich N. A. Abnet C. C. Al-Ghalith G. A. (2019). Reproducible, interactive, scalable and extensible microbiome data science using QIIME 2. *Nat. Biotechnol.* 37 852–857. 31341288 10.1038/s41587-019-0209-9 PMC7015180

[B11] Buitenhuis E. T. Li W. K. Vaulot D. Lomas M. W. Landry M. Partensky F. (2012). Picophytoplankton biomass distribution in the global ocean. *Earth Syst. Sci. Data* 4 37–46. 10.5194/essd-4-37-2012

[B12] Callahan B. J. McMurdie P. J. Rosen M. J. Han A. W. Johnson A. J. A. Holmes S. P. (2016). DADA2: high-resolution sample inference from Illumina amplicon data. *Nat. Methods* 13 581–583. 10.1038/nmeth.3869 27214047 PMC4927377

[B13] Celepli N. Sundh J. Ekman M. Dupont C. L. Yooseph S. Bergman B. (2017). Meta−omic analyses of Baltic Sea cyanobacteria: diversity, community structure and salt acclimation. *Environ. Microbiol.* 19 673–686. 10.1111/1462-2920.13592 27871145

[B14] Chen B. Liu H. Landry M. R. DaI M. Huang B. Sune J. (2009). Close coupling between phytoplankton growth and microzooplankton grazing in the western South China Sea. *Limnol. Oceanogr.* 54 1084–1097. 10.4319/lo.2009.54.4.1084

[B15] Chen B. Liu H. Xiao W. Wang L. Huang B. (2020). A machine-learning approach to modeling picophytoplankton abundances in the South China Sea. *Prog. Oceanogr.* 189:102456. 10.1016/j.pocean.2020.102456

[B16] Chen F. Wang K. Kan J. Suzuki M. T. Wommack K. E. (2006). Diverse and unique picocyanobacteria in Chesapeake Bay, revealed by 16S-23S rRNA internal transcribed spacer sequences. *Appl. Environ. Microbiol.* 72 2239–2243. 10.1128/AEM.72.3.2239-2243.2006 16517680 PMC1393199

[B17] Choi D. H. Noh J. H. (2009). Phylogenetic diversity of *Synechococcus* strains isolated from the East China Sea and the East Sea. *FEMS Microbiol. Ecol.* 69 439–448. 19624741 10.1111/j.1574-6941.2009.00729.x

[B18] Choi D. H. Noh J. H. Shim J. (2013b). Seasonal changes in picocyanobacterial diversity as revealed by pyrosequencing in temperate waters of the East China Sea and the East Sea. *Aquat. Microb. Ecol.* 71 75–90.

[B19] Choi D. H. Noh J. H. Lee J.-H. (2013a). Application of pyrosequencing method for investigating the diversity of *Synechococcus* subcluster 5.1 in open ocean. *Microbes Environ.* 29 17–22. 10.1264/jsme2.me13063 24389411 PMC4041225

[B20] Christie-Oleza J. A. Scanlan D. J. Armengaud J. (2015). “You produce while I clean up”, a strategy revealed by exoproteomics during *Synechococcus*–*Roseobacter* interactions. *Proteomics* 15 3454–3462. 10.1002/pmic.201400562 25728650 PMC4949626

[B21] Chung C. C. Chang J. Gong G. C. Hsu S. C. Chiang K. P. Liao C. W. (2011). Effects of Asian Dust Storms on *Synechococcus* populations in the subtropical Kuroshio Current. *Mar. Biotechnol.* 13 751–763. 10.1007/s10126-010-9336-5 21153675

[B22] Cottee-Jones H. E. W. Whittaker R. J. (2012). Perspective: the keystone species concept: a critical appraisal. *Front. Biogeogr.* 4:117–127.

[B23] Dong J. D. Zhang Y. Y. Zhang S. Wang Y. S. Yang Z. H. Wu M. L. (2010). Identification of temporal and spatial variations of water quality in Sanya Bay, China by three-way principal component analysis. *Environ. Earth Sci.* 60 1673–1682.

[B24] Dufresne A. Ostrowski M. Scanlan D. J. Garczarek L. Mazard S. Palenik B. P. (2008). Unraveling the genomic mosaic of a ubiquitous genus of marine cyanobacteria. *Genome Biol.* 9:R90. 10.1186/gb-2008-9-5-r90 18507822 PMC2441476

[B25] Farrant G. K. Doré H. Cornejo-Castillo F. M. Partensky F. Ratin M. Ostrowski M. (2016). Delineating ecologically significant taxonomic units from global patterns of marine picocyanobacteria. *Proc. Natl. Acad. Sci. U.S.A.* 113 E3365–E3374. 10.1073/pnas.1524865113 27302952 PMC4914166

[B26] Flombaum P. Gallegos J. L. Gordillo R. A. Rincón J. Martiny A. C. (2013). Present and future global distributions of the marine Cyanobacteria *Prochlorococcus* and *Synechococcus*. *Proc. Natl. Acad. Sci. U.S.A.* 110 9824–9829. 10.1073/pnas.1307701110 23703908 PMC3683724

[B27] Fucich D. Chen F. (2020). Presence of toxin-antitoxin systems in picocyanobacteria and their ecological implications. *ISME J.* 14 2843–2850. 10.1038/s41396-020-00746-4 32814864 PMC7784851

[B28] Fuller N. J. Marie D. Partensky F. Vaulot D. Post A. F. Scanlan D. J. (2003). Clade-specific 16S ribosomal DNA oligonucleotides reveal the predominance of a single marine *Synechococcus* clade throughout a stratified water column in the Red Sea. *Appl. Environ. Microbiol.* 69 2430–2443. 10.1128/AEM.69.5.2430-2443.2003 12732508 PMC154553

[B29] Gill A. S. Lee A. McGuire K. L. (2017). Phylogenetic and functional diversity of total (DNA) and expressed (RNA) bacterial communities in urban green infrastructure bioswale soils. *Appl. Environ. Microbiol.* 83:e00287-17. 10.1128/AEM.00287-17 28576763 PMC5541207

[B30] Huang S. Wilhelm S. W. Harvey H. R. Taylor K. Jiao N. Chen F. (2012). Novel lineages of *Prochlorococcus* and *Synechococcus* in the global oceans. *ISME J.* 6:285. 10.1038/ismej.2011.106 21955990 PMC3260499

[B31] Iturriaga R. Mitchell B. (1986). Chroococcoid cyanobacteria: a significant component in the food web dynamics of the open ocean. *Mar. Ecol. Prog. Ser.* 28 291–297.

[B32] Jing H. Zhang R. Pointing S. B. Liu H. Qian P. (2009). Genetic diversity and temporal variation of the marine *Synechococcus* community in the subtropical coastal waters of Hong Kong. *Can. J. Microbiol.* 55 311–318. 10.1139/w08-138 19370074

[B33] Kent A. G. Baer S. E. Mouginot C. Huang J. S. Larkin A. A. Lomas M. W. (2019). Parallel phylogeography of *Prochlorococcus* and *Synechococcus*. *ISME J.* 13 430–441. 30283146 10.1038/s41396-018-0287-6 PMC6331572

[B34] Li G. Song Q. Zheng P. Zhang X. Zou S. Li Y. (2021). Dynamics and distribution of marine *Synechococcus* abundance and genotypes during seasonal hypoxia in a coastal marine ranch. *J. Mar. Sci. Eng.* 9:549.

[B35] Ling J. Zhang Y. Dong J. Wang Y. Huang H. Chen L. (2013). Spatial variability of cyanobacterial community composition in Sanya Bay as determined by DGGE fingerprinting and multivariate analysis. *Chin. Sci. Bull.* 58 1019–1027.

[B36] Liu H. Jing H. Wong T. H. Chen B. (2014). Co−occurrence of phycocyanin−and phycoerythrin−rich *Synechococcus* in subtropical estuarine and coastal waters of Hong Kong. *Environ. Microbiol. Rep.* 6 90–99. 10.1111/1758-2229.12111 24596266

[B37] Mackey K. R. M. Hunter-Cevera K. Britten G. L. Murphy L. G. Sogin M. L. Huber J. A. (2017). Seasonal succession and spatial patterns of *Synechococcus* microdiversity in a salt marsh estuary revealed through 16S rRNA gene oligotyping. *Front. Microbiol.* 8:1496. 10.3389/fmicb.2017.01496 28848514 PMC5552706

[B38] Mazard S. Ostrowski M. Partensky F. Scanlan D. J. (2012). Multi-locus sequence analysis, taxonomic resolution and biogeography of marine *Synechococcus*. *Environ. Microbiol.* 14 372–386. 10.1111/j.1462-2920.2011.02514.x 21651684

[B39] Meixia Z. Kefu Y. Qiaomin Z. Qi S. (2008). Spatial pattern of coral diversity in Luhuitou fringing reef, Sanya, China. *Acta Ecol. Sin.* 28 1419–1428.

[B40] Mella-Flores D. Mazard S. Humily F. Partensky F. Mahé F. Bariat L. (2011). Is the distribution of *Prochlorococcus* and *Synechococcus* ecotypes in the Mediterranean Sea affected by global warming? *Biogeosciences* 8 2785–2804.

[B41] Moisan T. A. Blattner K. L. Makinen C. P. (2010). Influences of temperature and nutrients on *Synechococcus* abundance and biomass in the southern Mid-Atlantic Bight. *Cont. Shelf Res.* 30 1275–1282.

[B42] Mühling M. Fuller N. J. Somerfield P. J. Post A. F. Wilson W. H. Scanlan D. J. (2006). High resolution genetic diversity studies of marine *Synechococcus* isolates using *rpo*C1-based restriction fragment length polymorphism. *Aquat. Microb. Ecol.* 45 263–275.

[B43] Nagarkar M. Wang M. Valencia B. Palenik B. (2021). Spatial and temporal variations in *Synechococcus* microdiversity in the Southern California coastal ecosystem. *Environ. Microbiol.* 23 252–266. 10.1111/1462-2920.15307 33169926

[B44] Newman M. E. J. (2006). Modularity and community structure in networks. *Proc. Natl. Acad. Sci. U.S.A.* 103 8577–8582. 16723398 10.1073/pnas.0601602103 PMC1482622

[B45] Nguyen L. T. Schmidt H. A. Von Haeseler A. Minh B. Q. (2015). IQ-TREE: a fast and effective stochastic algorithm for estimating maximum-likelihood phylogenies. *Mol. Biol. Evol.* 32 268–274. 10.1093/molbev/msu300 25371430 PMC4271533

[B46] Paerl R. W. Turk K. A. Beinart R. A. Chavez F. P. Zehr J. P. (2012). Seasonal change in the abundance of *Synechococcus* and multiple distinct phylotypes in Monterey Bay determined by *rbc*L and *nar*B quantitative PCR. *Environ. Microbiol.* 14 580–593. 10.1111/j.1462-2920.2011.02594.x 21955724

[B47] Paerl R. Johnson K. Welsh R. Worden A. Chavez F. Zehr J. (2011). Differential distributions of *Synechococcus* subgroups across the California current system. *Front. Microbiol.* 2:59. 10.3389/fmicb.2011.00059 21833315 PMC3153035

[B48] Palenik B. Ren Q. Dupont C. L. Myers G. S. Heidelberg J. F. Badger J. H. (2006). Genome sequence of *Synechococcus* CC9311: insights into adaptation to a coastal environment. *Proc. Natl. Acad. Sci. U.S.A.* 103 13555–13559. 10.1073/pnas.0602963103 16938853 PMC1569201

[B49] Post A. F. Penno S. Zandbank K. Paytan A. Huse S. Mark Welch D. (2011). Long term seasonal dynamics of *Synechococcus* population structure in the Gulf of Aqaba, Northern Red Sea. *Front. Microbiol.* 2:131. 10.3389/fmicb.2011.00131 21734910 PMC3122069

[B50] Qiu D. Huang L. Zhang J. Lin S. (2010). Phytoplankton dynamics in and near the highly eutrophic Pearl River Estuary, South China Sea. *Cont. Shelf Res.* 30 177–186. 10.1016/j.marpolbul.2011.01.018 21316714

[B51] Qiu W. (2013). The Sanya coral reef national marine nature reserve, China: a governance analysis. *Mar. Policy* 41 50–56.

[B52] Rajaneesh K. Mitbavkar S. (2013). Factors controlling the temporal and spatial variations in *Synechococcus* abundance in a monsoonal estuary. *Mar. Environ. Res.* 92 133–143. 10.1016/j.marenvres.2013.09.010 24094891

[B53] Ramos C. Mølbak L. Molin S. (2000). Bacterial activity in the rhizosphere analyzed at the single-cell level by monitoring ribosome contents and synthesis rates. *Appl. Environ. Microbiol.* 66 801–809. 10.1128/AEM.66.2.801-809.2000 10653754 PMC91899

[B54] Robidart J. C. Preston C. M. Paerl R. W. Turk K. A. Mosier A. C. Francis C. A. (2012). Seasonal *Synechococcus* and *Thaumarchaeal* population dynamics examined with high resolution with remote *in situ* instrumentation. *ISME J.* 6 513–523. 10.1038/ismej.2011.127 21975596 PMC3280143

[B55] Salgar-Chaparro S. J. Machuca L. L. (2019). Complementary DNA/RNA-based profiling: characterization of corrosive microbial communities and their functional profiles in an oil production facility. *Front. Microbiol.* 10:2587. 10.3389/fmicb.2019.02587 31787960 PMC6853844

[B56] Schmidt K. Birchill A. J. Atkinson A. Brewin R. J. Clark J. R. Hickman A. E. (2020). Increasing picocyanobacteria success in shelf waters contributes to long−term food web degradation. *Glob. Change Biol.* 26 5574–5587. 10.1111/gcb.15161 32506810

[B57] Segata N. Izard J. Waldron L. Gevers D. Miropolsky L. Garrett W. S. (2011). Metagenomic biomarker discovery and explanation. *Genome Biol.* 12:R60. 10.1186/gb-2011-12-6-r60 21702898 PMC3218848

[B58] Six C. Thomas J.-C. Garczarek L. Ostrowski M. Dufresne A. Blot N. (2007). Diversity and evolution of phycobilisomes in marine *Synechococcus* spp.: a comparative genomics study. *Genome Biol.* 8:R259. 10.1186/gb-2007-8-12-r259 18062815 PMC2246261

[B59] Sohm J. A. Ahlgren N. A. Thomson Z. J. Williams C. Moffett J. W. Saito M. A. (2016). Co-occurring *Synechococcus* ecotypes occupy four major oceanic regimes defined by temperature, macronutrients and iron. *ISME J.* 10:333. 10.1038/ismej.2015.115 26208139 PMC4737926

[B60] Steele J. A. Countway P. D. Xia L. Vigil P. D. Beman J. M. Kim D. Y. (2011). Marine bacterial, archaeal and protistan association networks reveal ecological linkages. *ISME J.* 5 1414–1425. 10.1038/ismej.2011.24 21430787 PMC3160682

[B61] Steven B. Hesse C. Soghigian J. Gallegos-Graves L. V. Dunbar J. (2017). Simulated rRNA/DNA ratios show potential to misclassify active populations as dormant. *Appl. Environ. Microbiol.* 83:e0696-17. 10.1128/AEM.00696-17 28363969 PMC5440720

[B62] Tai V. Palenik B. (2009). Temporal variation of *Synechococcus* clades at a coastal Pacific Ocean monitoring site. *ISME J.* 3 903–915. 10.1038/ismej.2009.35 19360028

[B63] Titlyanov E. A. Titlyanova T. V. Scriptsova A. V. Ren Y. Li X. Huang H. (2019). Interannual and seasonal changes in the benthic algae flora of coral reef in Xiaodong Hai (Hainan Island, China). *J. Mar. Sci. Eng.* 7:243.

[B64] Tsai A. Y. Chiang K. P. Chan Y. F. Lin Y. C. Chang J. (2006). Pigmented nanoflagellates in the coastal western subtropical Pacific are important grazers on *Synechococcus* populations. *J. Plankton Res.* 29 71–77.

[B65] van den Engh G. J. Doggett J. K. Thompson A. W. Doblin M. A. Gimpel C. N. G. Karl D. M. (2017). Dynamics of *Prochlorococcus* and *Synechococcus* at sation ALOHA revealed through flow cytometry and high-resolution vertical sampling. *Front. Mar. Sci.* 4:359. 10.3389/fmars.2017.00359

[B66] Wang K. Wommack K. E. Chen F. (2011). Abundance and distribution of *Synechococcus* spp. and cyanophages in the Chesapeake Bay. *Appl. Environ. Microbiol.* 77 7459–7468. 10.1128/AEM.00267-11 21821760 PMC3209163

[B67] Wang T. Chen X. Qin S. Li J. (2021a). Phylogenetic and phenogenetic diversity of *Synechococcus* along a Yellow Sea section reveal its environmental dependent distribution and co-occurrence microbial pattern. *J. Mar. Sci. Eng.* 9:1018.

[B68] Wang T. Chen X. Li J. Qin S. (2021b). Distribution and phenogenetic diversity of *Synechococcus* in the Bohai Sea, China. *J. Oceanol. Limnol.* 39 1–13.

[B69] Watts D. J. Strogatz S. H. (1998). Collective dynamics of ‘small-world’networks. *Nature* 393 440–442. 9623998 10.1038/30918

[B70] Wei Y. Sun J. Zhang X. Wang J. Huang K. (2019). Picophytoplankton size and biomass around equatorial eastern Indian Ocean. *Microbiologyopen* 8:e00629. 10.1002/mbo3.629 29656564 PMC6391267

[B71] Xia X. Cheung S. Endo H. Suzuki K. Liu H. (2019). Latitudinal and vertical variation of *Synechococcus* assemblage composition along 170^°^ w transect from the South Pacific to the Arctic Ocean. *Microb. Ecol.* 77 333–342. 10.1007/s00248-018-1308-8 30610255

[B72] Xia X. Guo W. Tan S. Liu H. (2017a). *Synechococcus* assemblages across the salinity gradient in a salt wedge estuary. *Front. Microbiol.* 8:1254. 10.3389/fmicb.2017.01254 28729864 PMC5498518

[B73] Xia X. Partensky F. Garczarek L. Suzuki K. Guo C. Yan Cheung S. (2017b). Phylogeography and pigment type diversity of *Synechococcus* cyanobacteria in surface waters of the northwestern Pacific Ocean. *Environ. Microbiol.* 19 142–158. 10.1111/1462-2920.13541 27668842

[B74] Xia X. Vidyarathna N. K. Palenik B. Lee P. Liu H. (2015). Comparison of the seasonal variations of *Synechococcus* assemblage structures in estuarine waters and coastal waters of Hong Kong. *Appl. Environ. Microbiol.* 81 7644–7655. 10.1128/AEM.01895-15 26319880 PMC4592875

[B75] Zhang C. Huang H. Ye C. Huang L. Li X. Lian J. (2013). Diurnal and seasonal variations of carbonate system parameters on Luhuitou fringing reef, Sanya Bay, Hainan Island, South China Sea. *Deep Sea Res. Part II Top. Stud. Oceanogr.* 96 65–74. 10.1016/j.dsr2.2013.02.013

[B76] Zhang X. Shi Z. Ye F. Zeng Y. Huang X. (2013). Picophytoplankton abundance and distribution in three contrasting periods in the Pearl River Estuary, South China. *Mar. Freshw. Res.* 64 692–705. 10.1071/mf12303

[B77] Zwirglmaier K. Heywood J. L. Chamberlain K. Woodward E. M. S. Zubkov M. V. Scanlan D. J. (2007). Basin−scale distribution patterns of picocyanobacterial lineages in the Atlantic Ocean. *Environ. Microbiol.* 9 1278–1290. 10.1111/j.1462-2920.2007.01246.x 17472640

[B78] Zwirglmaier K. Jardillier L. Ostrowski M. Mazard S. Garczarek L. Vaulot D. (2008). Global phylogeography of marine *Synechococcus* and *Prochlorococcus* reveals a distinct partitioning of lineages among oceanic biomes. *Environ. Microbiol.* 10 147–161. 10.1111/j.1462-2920.2007.01440.x 17900271

