# Supplementary material for: Spatio-Temporal Variation of Synechococcus Assemblages at DNA and cDNA Levels in the Tropical Estuarine and Coastal Waters
Source: Front Microbiol. 2022 Mar 3;13:837037. doi: 10.3389/fmicb.2022.837037 (PMC8928118; doi:10.3389/fmicb.2022.837037)
Supplement: Supplementary file 1 [file Data_Sheet_1.docx]

Supplementary Material

# Supplementary Figures and Tables

## Supplementary Figures


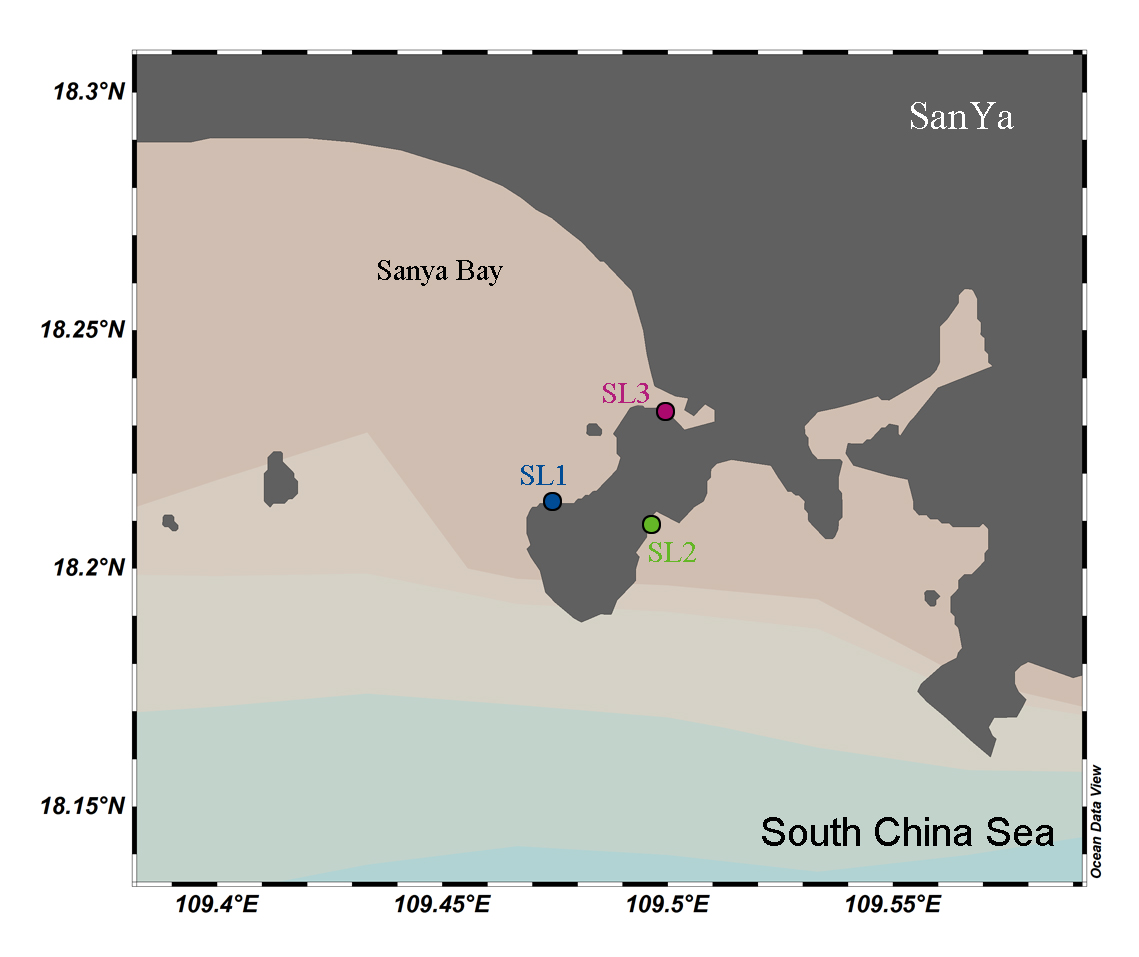


**Supplementary Figure S1.** The geographic location of three sampling stations. Stns. SL1 and SL2 represented coastal stations and stn. SL3 represented estuarine station.


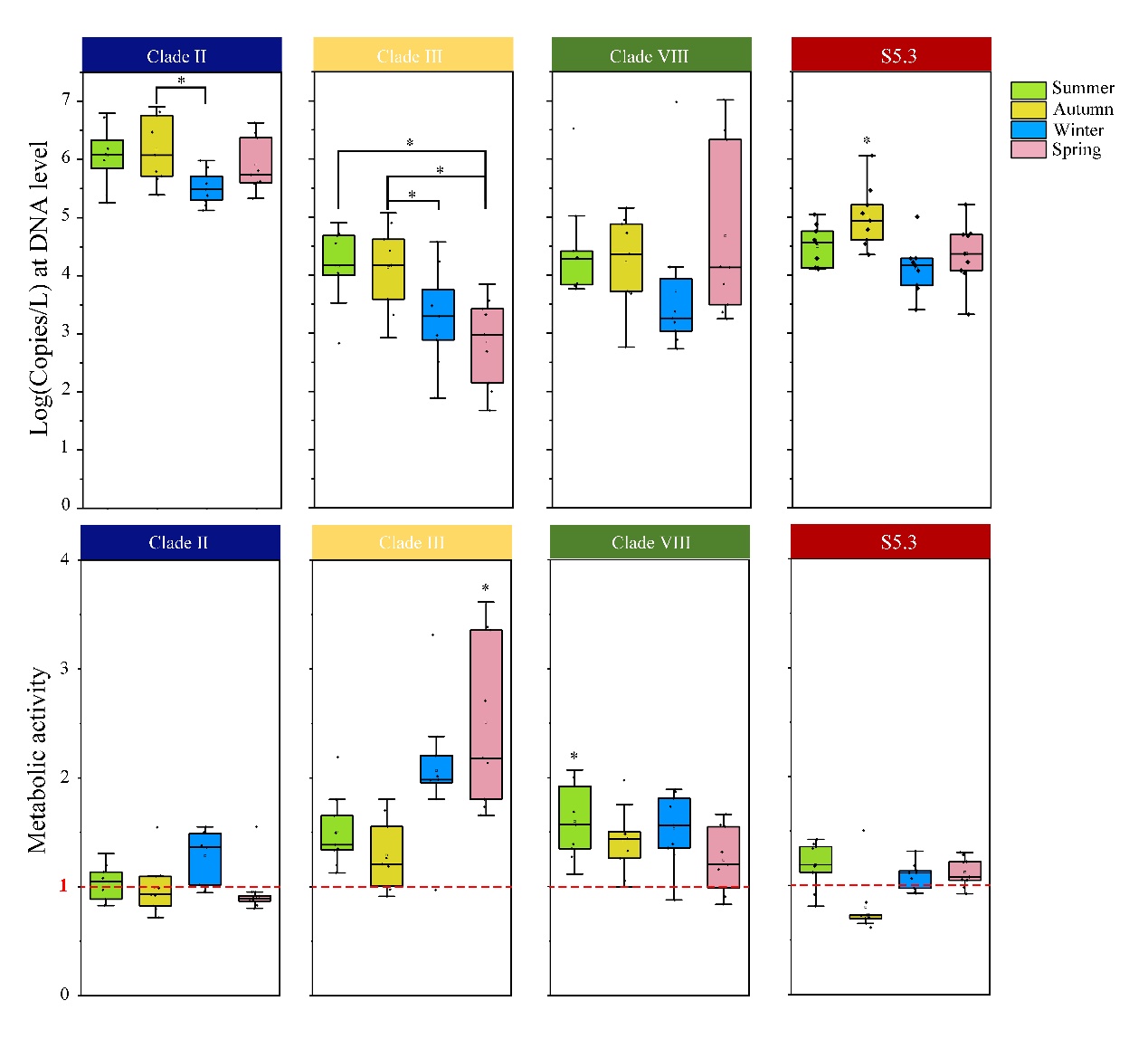


**Supplementary Figure S2.** Box plots showing the temporal variation of (up panel) gene abundances and (bottom panel) metabolic activity reflected by gene transcript abundance/gene abundance ratios of five clades. * – *p* < 0.05; ** – *p* < 0.01; Summer – Jun. to Aug.; Autumn – Sep. to Nov.; Spring – Dec. to Feb.; Winter – Mar. to May.


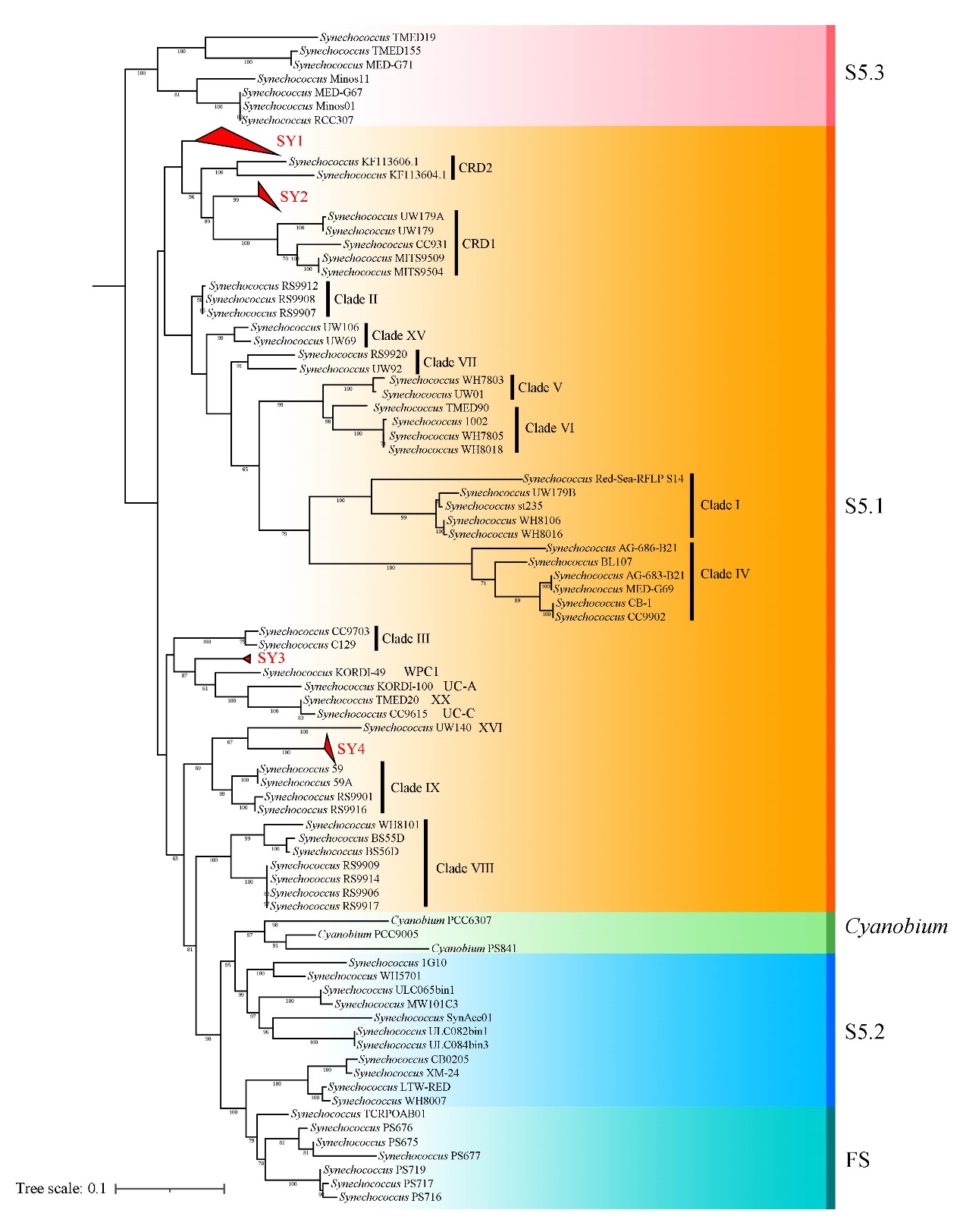


**Supplementary Figure S3** The maximum-likelihood phylogenetic tree based on the *rpo*C1 gene sequence of unclassified ASVs.

## Supplementary Table

**Supplementary Table S1** Network parameters of *Synechococcus* community.^1^

| **Network parameters** | **DNA level** | **cDNA level** |
| --- | --- | --- |
| Positive correlation | 13,387 | 23,078 |
| Negative correlation | 1,608 | 3,477 |
| Number of edges | 14,995 | 26,555 |
| Number of nodes | 523 | 440 |
| Average degree | 59.34 | 122.71 |
| Average path length | 2.24 | 1.85 |
| Clustering coefficient | 0.523 | 0.743 |
| Modularity | 0.653 | 0.621 |
| Number of sub-communities: | 4 | 3 |
| Phylogenetic affiliations of top three ASVs ranked by closeness centrality | FS/S5.2 | S5.3 |
|  | FS/S5.2 | SY3 |
|  | FS/S5.2 | WPC1 |

^1^ Average degree – the average number of edges incident to/from each node; Average path length – the average shortest path between two nodes; Clustering coefficient – the average number of triangles in which node participates normalized by the maximum possible number of such triangles; Modularity – a topological parameter judging the merits and demerits of the results of sub-community division.
